# 20(*S*)-Protopanaxadiol Phospholipid Complex: Process Optimization, Characterization, In Vitro Dissolution and Molecular Docking Studies

**DOI:** 10.3390/molecules21101396

**Published:** 2016-10-19

**Authors:** Yiqiong Pu, Xitong Zhang, Qi Zhang, Bing Wang, Yuxi Chen, Chuanqi Zang, Yuqin Wang, Tina Ting-Xia Dong, Tong Zhang

**Affiliations:** 1 Experiment Center for Teaching and Learning, Shanghai University of Traditional Chinese Medicine, No. 1200 Cailun Road, Pudong New District, Shanghai 201203, China; puyiq@163.com; 2 School of Pharmacy, Shanghai University of Traditional Chinese Medicine, No. 1200 Cailun Road, Pudong New District, Shanghai 201203, China; zhangxitong1990@126.com (X.Z.); zhang645654@163.com (Q.Z.); yuxi59shutcm@163.com (Y.C.); zangchuanqi1991@sina.com (C.Z.); 3 Zhejiang BioAsia Institute of Life Science, No. 1938 Xinqun Road, Economic and Technical Development Zone, Pinghu 314200, China; wyq@bioasia.com.cn; 4 Division of Life Science and Center for Chinese Medicine, The Hong Kong University of Science and Technology, Clear Water Bay Road, Hong Kong, China; botina@ust.hk

**Keywords:** 20(*S*)-protopanaxadiol, phospholipid complex, central composite design, DSC, molecular docking

## Abstract

20(*S*)-Protopanaxadiol (PPD), a bioactive compound extracted from ginseng, possesses cardioprotective, neuroprotective, anti-inflammatory, antiestrogenic, anticancer and anxiolytic effects. However, the clinical application of PPD is limited by its weak aqueous solubility. In this study, we optimized an efficient method of preparing its phospholipid complex (PPD-PLC) using a central composite design and response surface analysis. The prepared PPD-PLC was characterized by differential scanning calorimetric, powder X-ray diffraction, Fourier-transformed infrared spectroscopy and nuclear magnetic resonance analyses associated with molecular docking calculation. The equilibrium solubility of PPD-PLC in water and *n*-octanol increased 6.53- and 1.53-times, respectively. Afterwards, using PPD-PLC as the intermediate, the PPD-PLC-loaded dry suspension (PPD-PLC-SU) was prepared with our previous method. In vitro evaluations were conducted on PPD-PLC and PPD-PLC-SU, including dissolution behaviors and stability properties under different conditions. Results of in vitro dissolution behavior revealed the improved dissolution extents and rates of PPD-PLC and PPD-PLC-SU (*p* < 0.05). Results of the formulation stability investigation also exposed the better stability of PPD-PLC-SU compared with free PPD. Therefore, phospholipid complex technology is a useful formulation strategy for BCS II drugs, as it could effectively improve their hydrophilicity and lipophilicity.

## 1. Introduction

**Figure 1:**
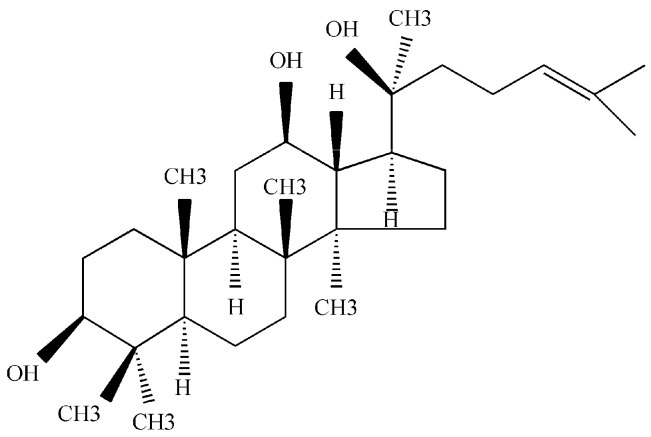
Structure of 20(*S*)-protopanaxadiol (PPD).

Ginseng, one of the most popular phytomedicines, has been widely used in medical practice for thousands of years in East Asia. Ginsenosides are reported to be the major pharmacologically-active components of ginseng [1,2,3,4]. 20(*S*)-Protopanaxadiol (PPD; Figure 1), a major aglycone of many bioactive ginsenosides that include ginsenoside Rb_1_, Rb_2_, Rb_3_, Rc, Rd, Rg_3_ and Rh_2_, is the metabolite resulting from the loss of the attached glucosyl group(s) in the gastrointestinal tract through a series of deglycosylation steps [5]. PPD has a series of extensive pharmacological actions, including cardioprotection [6,7], neuroprotection [8], anti-inflammation [9], antiestrogen [10,11], anticancer (including breast [12], prostate [13,14], ovarian cancers [15] and malignant glioblastoma multiforme [16]) and anxiolytic [17].

However, PPD is a chemical Biopharmaceutics Classification System (BCS) II drug with inferior water solubility and favorable permeability. Our previous study on the properties of free PPD also revealed its relatively favorable oil/water partition coefficient [18]. Moreover, other pharmacokinetic studies on PPD have reported that its low oral bioavailability (approximately 29.39%) is related to its inferior oral absorption and extensive metabolism by cytochrome P450 [18,19,20]. These pharmaceutically-unfavorable properties of PPD have greatly hindered its therapeutic application and development as a promising clinical agent; thus, enhancing its solubility is critical for enhancing its bioavailability. In recent years, several formulations, including nanocrystal [21], nanoparticles [22], nanosuspensions [23], cubic nanoparticles [22] and micelles [24], have been developed to increase its oral bioavailability and expand its clinical application, as reported in the field of pharmaceutics. Moreover, our formulation studies on PPD have involved a self-microemulsifying drug delivery system (SMEDDS) [25], tablets [26], microspheres [27,28] and hydroxyapatite assemblies [29] in the past five years, all of which effectively improved PPD bioavailability or presented sustained dissolution properties.

In this pharmaceutical study, we predominantly focused on a phospholipid complex-based drug delivery system [30,31,32,33,34], which is expected to progressively influence the limitations that are associated with inferior drug solubility. Phospholipids are compound lipids derived from either glycerol (phosphor glycerides) or sphingosine (sphingomyelin) and capable of enhancing the solubility of drugs when used as biofunctional carriers. Moreover, the favorable biocompatibility of these phospholipids allows them to penetrate the cell membranes and enter the cytoplasm of living cells without disrupting the cellular lipid bilayers, and therefore, the complexation of drugs with phospholipids can improve their permeation and oral absorption, thereby enhancing their bioavailabilities [35] or pharmacological effects [36,37]. Recent successful cases in promoting the absorption of varied natural substances with phospholipids complex technology include those of cucurbitacin B [38], polyphenols [39], echinacoside [40], probucol [41], resveratrol [42] and tamoxifen [43]. However, because of its relatively low stability, the phospholipid complex (PLC) is typically used as a pharmaceutical intermediate in dosage forms. Therefore, phospholipid complexes are also associated with other drug delivery systems, such as nanoparticles [44,45], nanoemulsions [46], microparticles [34] and self-nanoemulsifying systems [47]. One of the common formulations with good dispersibility includes a dry suspension, wherein drug agents exist as a dispersion of particles formed through a simple preparation process with favorable dispersibility, rapid gastrointestinal absorption and consequently improved bioavailability [48].

Therefore, this study has optimized an efficient method to prepare a PPD-loaded phospholipid complex (PPD-PLC), using a central composite design (CCD) and response surface analysis (RSA). The produced PPD-PLC was characterized with differential scanning calorimetric (DSC), powder X-ray diffraction (PXRD), Fourier-transformed infrared spectroscopy (FTIR) and nuclear magnetic resonance (NMR) analyses, associated with molecular docking calculation. Equilibrium solubility was determined to compare the performance of PPD and PPD-PLC in hydrophilicity and lipophilicity. Afterwards, by using PPD-PLC as the intermediate, the PPD-PLC-loaded dry suspension (PPD-PLC-SU) was prepared using our previous method [49]. In vitro dissolution behaviors and stability properties under different conditions of PPD-PLC and PPD-PLC-SU were investigated, respectively.

## 2. Results

### 2.1. PPD-PLC Preparation

#### 2.1.1. Response Surface Analysis

Entrapment efficiency (EE, %) of PLC refers to the percentage of the entrapped drugs in the total added drugs when preparing PLC. After the scheduled experiments of CCD, the EE of the 20 batches is shown in Table 1, which varied from 45.33%–66.55%. Two model fitting equations were obtained using the statistical software Design-Expert (Version 8.0.6, Stat-Ease Inc., Minneapolis, MN, USA). The quadratic model fitting (Equation (2), *R*^2^ = 0.8279) was superior to the linear model (Equation (1), *R*^2^ = 0.5716) in terms of correlation coefficient (*R*^2^). Consequently, the quadratic model was selected as the optimal fitting model and was further validated using an *F*-test for the analysis of variance (ANOVA) to evaluate the significance of the regression (Table 2), which revealed a significant difference in the quadratic model (*F* > *F*_critical_, *p* < 0.05).

*Y* = 39.08694 + 10.49102*X*_1_ + 0.28773*X*_2_ + 0.58972*X*_3_ (*R*^2^ = 0.5716) (1)

*Y* = 65.50631 − 0.77977*X*_1_ − 0.47802*X*_2_ − 10.91742*X*_3_ + 0.29467*X*_1_*X*_2_ + 1.49000*X*_1_*X*_3_ − 0.22700*X*_2_*X*_3_ + 1.24698*X*_1_^2^ + 0.043137*X*_2_^2^ + 2.08036*X*_3_^2^ (*R*^2^ = 0.8279) (2)

**Figure 2:**
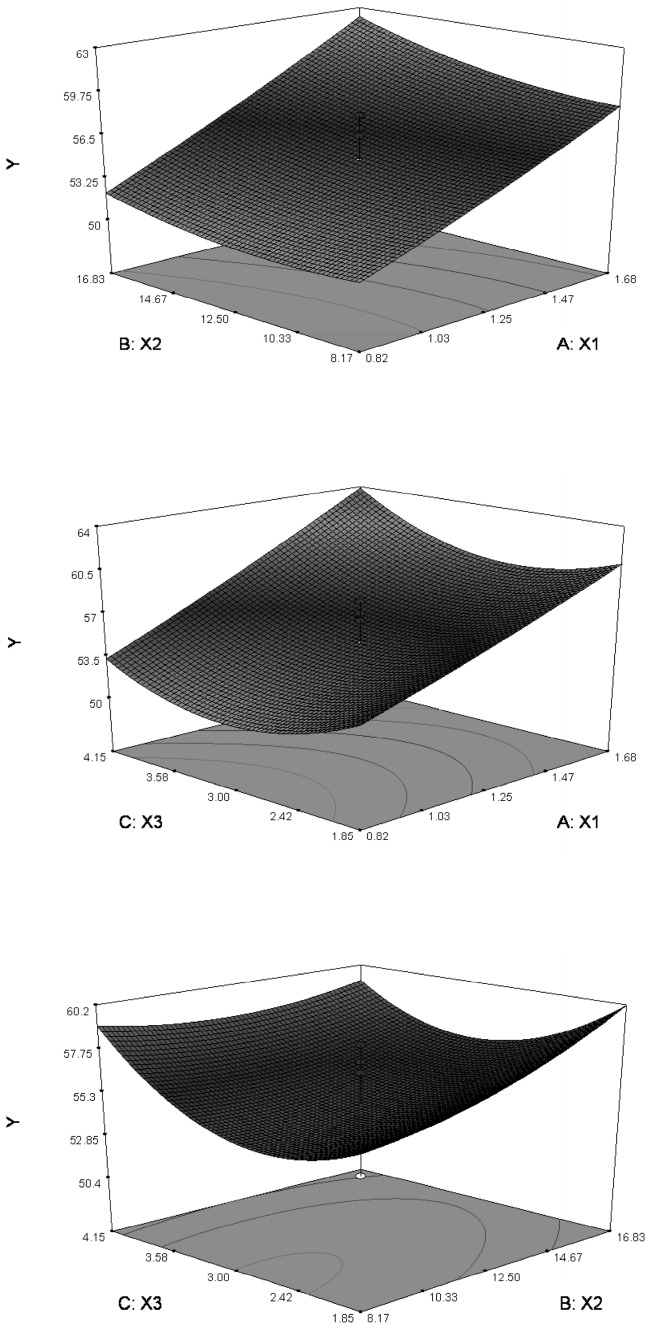
Response surface and contour plots based on entrapment efficiency (*Y*, %) as a function of the investigated factors: phospholipid-to-drug ratio (*X*_1_, g/g), reaction concentration of PPD (*X*_2_, mg/mL) and reaction time (*X*_3_, h).

The relationship between variables and the response could be visualized using response surface analysis RSA in Design-Expert, which generated the response surface and contour plots. As shown in Figure 2, a high level of *X*_1_ was found to be favorable to gain high EE. An increase in the *X*_3_ level caused the EE to increase initially and to decrease subsequently. However, the level of *X_2_* was observed to have little effect on the variation of EE. In the largest area of the *Y* value (EE, %) formed by the three-dimensional effects of response surfaces, the optimal process parameters were obtained as follows: phospholipid-to-drug ratio (*X*_1_, g/g) = 2:1 and reaction time (*X*_3_, h) = 5 h. The reaction concentration of PPD (*X*_2_, mg/mL) was set to 20 mg/mL to increase drug load capacity.

#### 2.1.2. Validation of Model Optimization

PPD-PLC was prepared under the optimized described parameters (*X*_1_, *X*_2_ and *X*_3_ were set to 2:1, 20 mg/mL and 5 h, respectively) to evaluate the optimization capability of the generated models derived from the circumscribed CCD and RSD results. The entrapment efficiency obtained with the predicted model was 78.04%, whereas that in PPD-PLC prepared in optimized conditions was 65.94% ± 4.02% (*n* = 3), with a −15.5% bias.

#### 2.1.3. Method Validation of Content Determination

Drug loading (DL, %) refers to the percentage of the drugs in PLC. In this study, EE and DL were determined by detecting the content of PPD in the complex products of PPD-PLC. Method validation included assessments of linearity, sensitivity, accuracy and precision. The linearity investigation results showed that the regression equation of calibration curves was *y* = 12.9*x* + 96.6 (*r*^2^ = 0.9999). The analytes showed linear behavior in the concentration range of 15.2–1015.6 μg/mL. The limit of detection (LOD) and the limit of quantification (LOQ) were determined by the signal-to-noise method to be 0.3 (*S*/*N* = 3) and 1.0 μg/mL (*S*/*N* = 10), respectively. The average recoveries were 98.2%, 100.4% and 96.0% (RSD = 1.4%, 1.7% and 0.7%, respectively) at the three spike levels of 120%, 100% and 80%, respectively. The results of precision with the standards at different levels (15.2, 162.5 and 1015.6 μg/mL) validated a good precision in intra-day (*n* = 6, RSD = 1.1%, 1.2% and 0.6%) and inter-day (*n* = 6, RSD = 4.6%, 2.3% and 1.9%) investigations. All of these results conformed to the standard recommended by ICH (Internation Conference on Harmonizaiton of Technical Requirements for Registration of Pharmaceuticals for Human Use) [50]. Therefore, the developed method for the determination of PPD in PPD-PLC can be considered sufficiently accurate, precise and fit for DL or EE determination in the optimization process.

### 2.2. Physiochemical Characterization of PPD-PLC

#### 2.2.1. Differential Scanning Calorimetry

**Figure 3:**
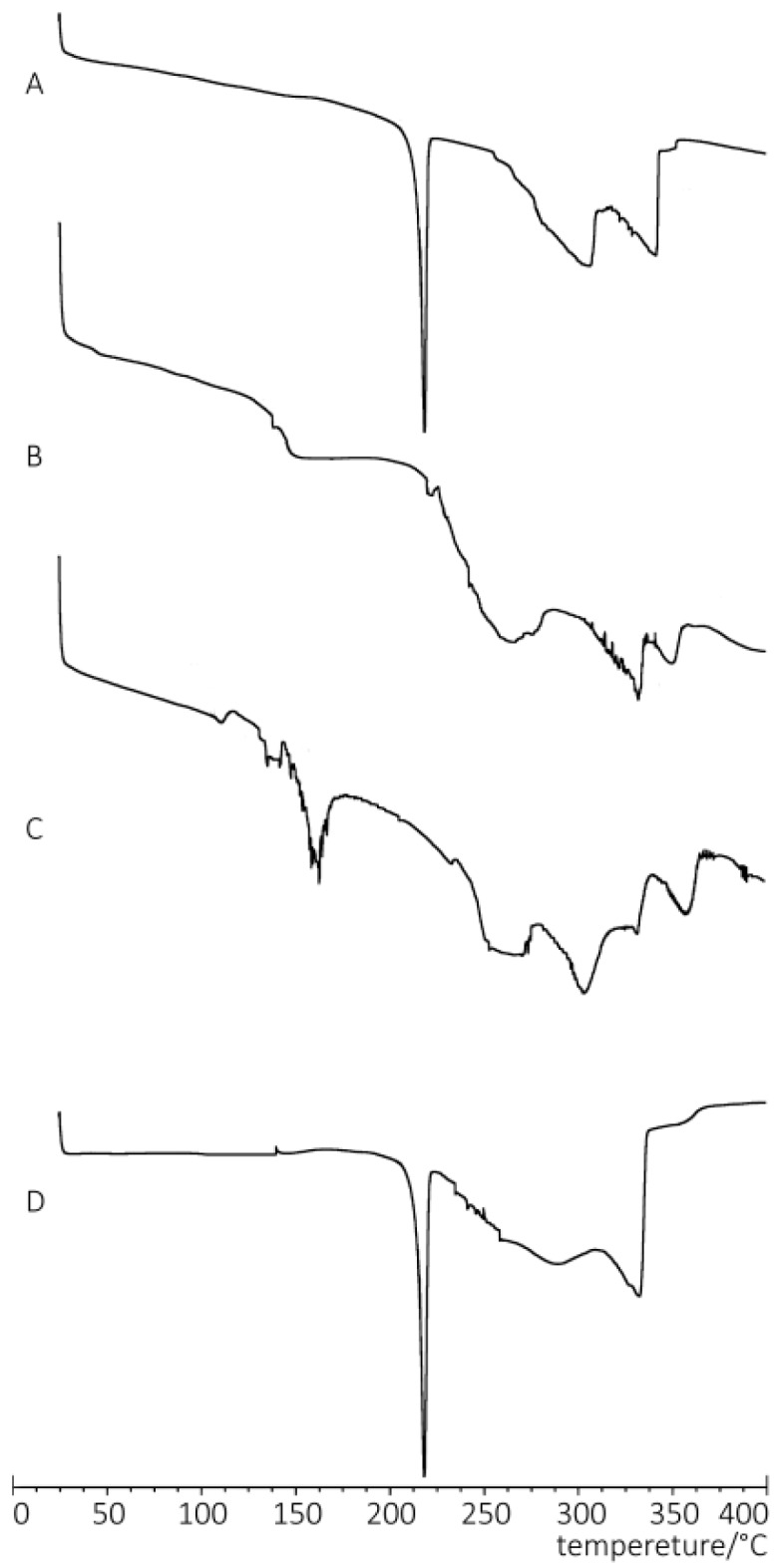
DSC thermograms of: (**A**) free PPD; (**B**) phospholipids; (**C**) PPD-phospholipid complex (PLC); and (**D**) physical mixture of PPD and phospholipids.

DSC analysis, a convenient method to investigate compatibility, provided information on the interaction between drug and excipients. The DSC thermograms of free PPD, phospholipids, PPD-PLC and the physical mixture of PPD and phospholipids are shown in Figure 3. The thermogram of the phospholipid exhibited two different endotherms in the range of 100–250 °C. The first endotherm at 137.16 °C was mild, which indicated that the formation of this peak was caused by hot movements of the polar part of the phospholipid molecule. The other endotherm at 218.47 °C might be related to the phase transition from a gel-like to a liquid-crystal state and the possible melting, isomeric or crystal changes of the carbon-hydrogen chain in the phospholipid [51]. It could be found that there was an endothermic peak at 330 °C in the DSC thermogram of phospholipid, which also appeared in the thermogram of the physical mixture, which confirmed that there was no interaction between PPD and phospholipid in their physical mixture during the DSC analysis. The thermogram of free PPD exhibited a sharp endothermal peak at 214.14 °C, corresponding to the melting point and crystalline state of free PPD with high purity; whereas the physical mixture exhibited a minor endothermic peak at 213.66 °C. The reason for this difference may be that the interaction between PPD and the phospholipid led to a lower melting point of the mixture than that of the free PPD, which was due to the increase in temperature. However, the DSC thermogram of PPD-PLC showed that the characteristic peaks of PPD and phospholipid disappeared. There were some apparent interactions between PPD and the phospholipid, such as the formation of hydrogen bonds and van der Waals forces.

#### 2.2.2. Powder X-ray Diffraction

**Figure 4:**
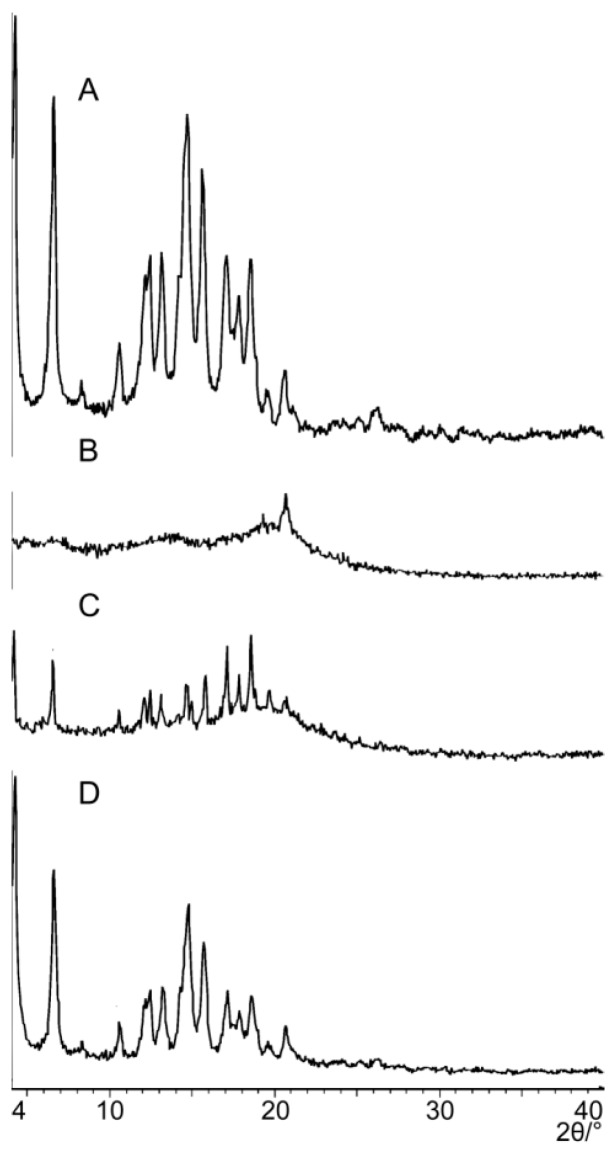
PXRD patterns of: (**A**) free PPD; (**B**) phospholipids; (**C**) PPD-PLC; and (**D**) physical mixture of PPD and phospholipids.

PXRD is often used to evaluate the existing form of PPD in the PPD-PLC. Figure 4 depicts the PXRD patterns of free PPD, phospholipids, PPD-PLC and the physical mixture. The free PPD PXRD pattern, revealing ten characteristic diffraction peaks, indicated that it was of superior crystallinity. The phospholipid showed only a wide band in its PXRD pattern without any crystalline peaks, which indicated that it was amorphous. In the diffraction pattern of the physical mixture, nine diffraction peaks were observed at 6.4, 10.5, 12.3, 13.1, 14.6, 15.6, 17.1, 18.5 and 20.6 (2θ), with positions and intensities consistent with that of free PPD. In the PPD-PLC diffraction pattern, the intensities of most characteristic peaks of PPD were reduced significantly, compared with the pattern of free PPD. This indicated that the crystallinity of PPD in PPD-PLC was decreased; and that most PPD existed in an amorphous form in the PPD-PLC, with a minority in the crystalline state.

#### 2.2.3. Fourier-Transformed Infrared Spectroscopy

**Figure 5:**
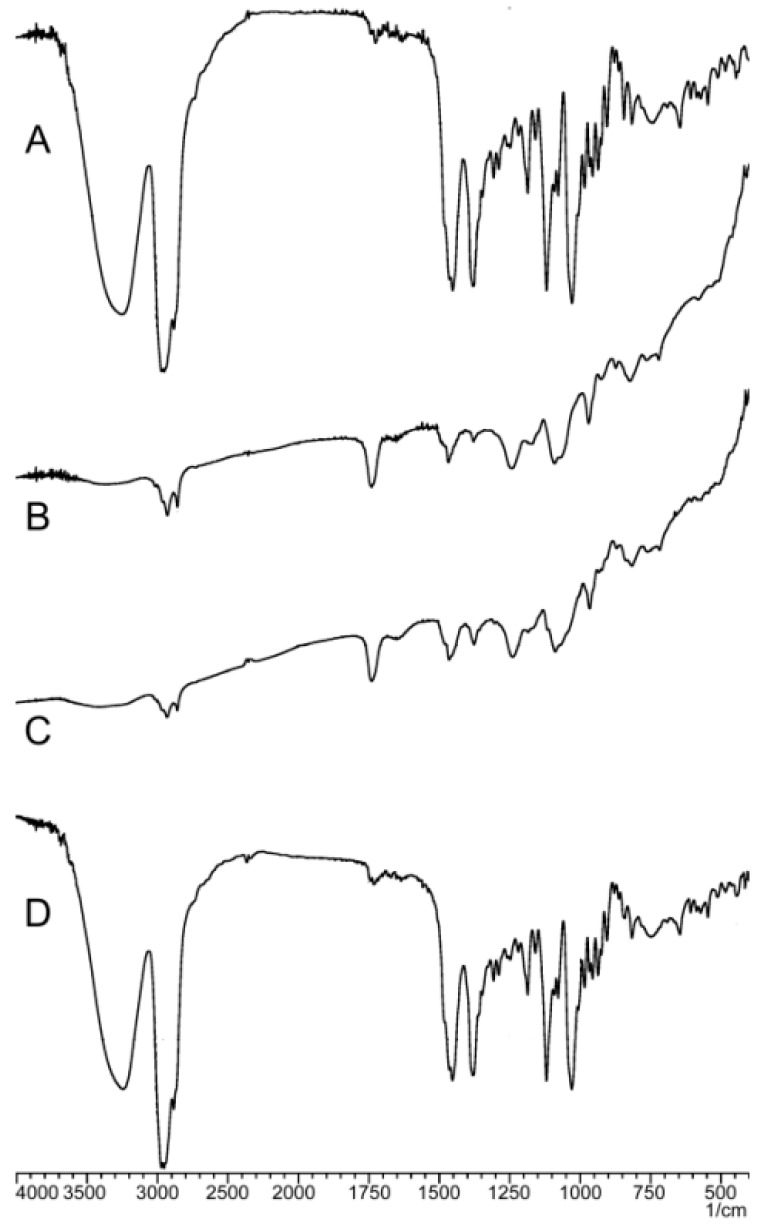
FTIR spectra of: (**A**) free PPD; (**B**) phospholipids; (**C**) PPD-PLC; and (**D**) physical mixture of PPD and phospholipids.

As shown in Figure 5, the characteristic absorptions of free PPD are presented at 3238.48 (–OH, υ), 2962.66, 2943.37, 2875.86 (–R, υ), 1452.40, 1381.03 (–CH_3_, υ), 1186.22, 1120.64, 1029.99 (C–O, υ) and 648.08 cm^−1^ (=C–H, υ). The wide absorption peaks at 3238.48 cm^−1^ suggested the formation of hydrogen bonds between molecules caused by the hydroxyl groups. The characteristic absorptions of phospholipids were observed at 2922.16, 2852.72, 1737.86 (C=O, υ), 1467.83 (=C–H, υ), 1242.16 (P=O, υ), 1091.71 (O–C, υ), 970.19 (P–O, υ) and 823.60 cm^−1^ (–C=CH, δ), which correspondingly indicated the presence of saturated alkyl, ester, phosphate and methyl groups. The spectrum of the physical mixture was the superposition of functional group peaks from the two compounds with the same peak positions. In the spectrum of PPD-PLC, the wide peak at 3238.48 cm^−1^ was shifted to 3207.62 cm^−1^ with a weakened intensity. The stretching vibration absorption peaks at 1186.22 and 1120.64 cm^−1^ (C–O, υ) disappeared, which illustrated that the hydroxyl groups might be influenced by the polar groups. C–H stretching vibration from the alkyl group only appeared at 2924.09 and 2852.72 cm^−1^.

##### 2.2.4. ^1^H-Nuclear Magnetic Resonance

**Figure 6:**
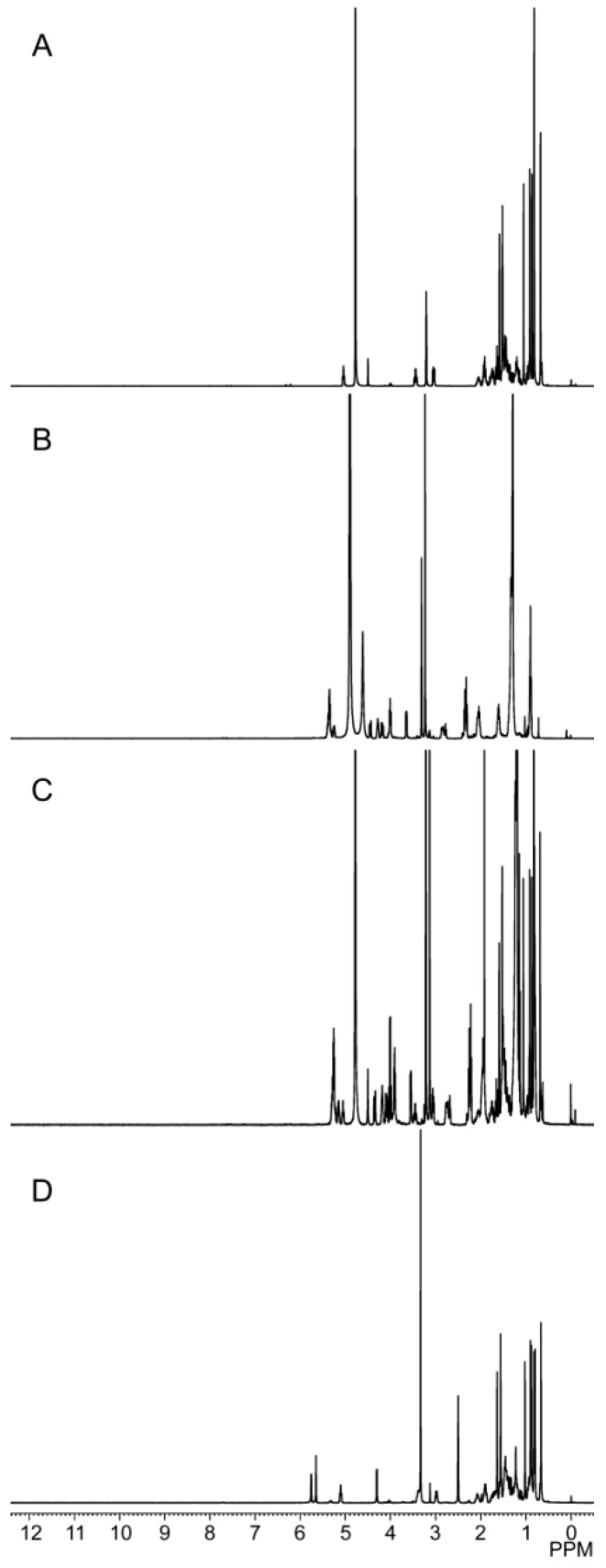
^1^H-NMR spectra of: (**A**) free PPD; (**B**) phospholipids; (**C**) PPD-PLC; and (**D**) physical mixture of PPD and phospholipids.

The spectra of the samples shown in Figure 6 contained the hydrogen signal peaks from PPD and phospholipid molecules. In the PPD spectrum, eight signals (δ = 0.82, 0.88, 0.92, 0.93, 1.05, 1.18, 1.47, 1.58 ppm) of H corresponding to eight methyl groups of PPD within the range 0.82–1.58 ppm were observed, along with the signal of H at 5.04 ppm representing the olefinic carbon of PPD. These characteristic signals of PPD were also observed in the PPD-PLC spectrum, where no change in the chemical shifts and shapes took place, compared with the spectra of PPD and phospholipids.

### 2.3. Molecular Docking

**Figure 7:**
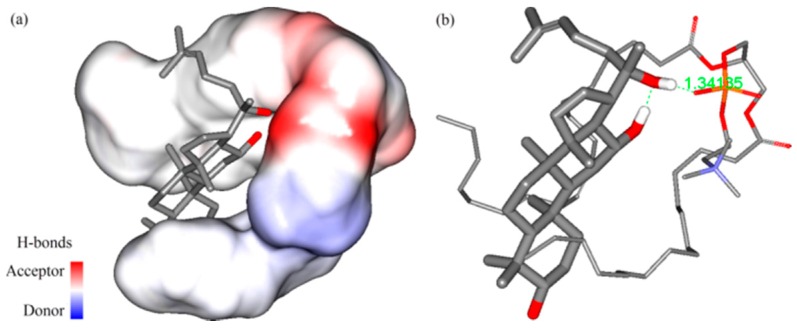
Initial molecular structures in the calculation. (**a**) Phospholipid; (**b**) PPD.

**Figure 8:**
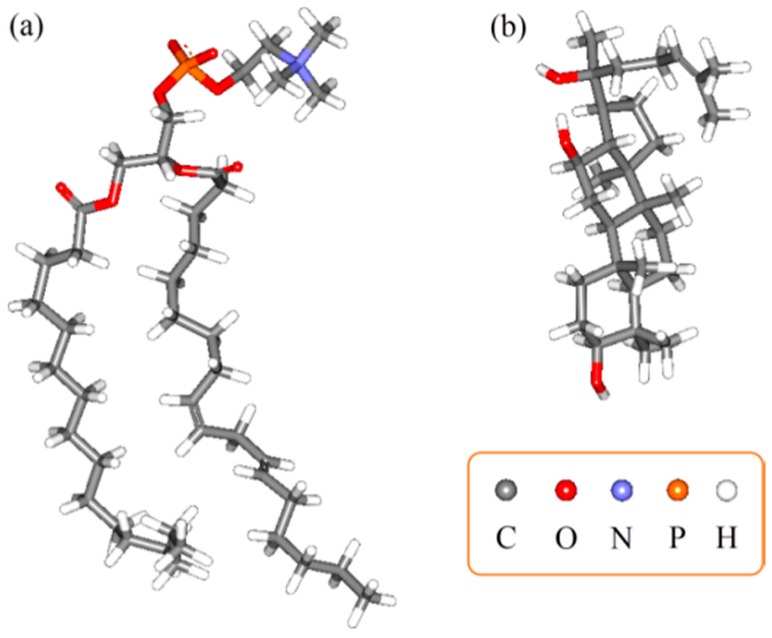
3D conformation of PPD and phospholipid after docking and optimization. (**a**) PPD and phospholipid with interpolated charge surface; (**b**) H-bond interactions in the complex.

The molecular modeling method can explore the mechanism of interaction on a molecular level. The best interaction sites of PPD and phospholipid could be seen in a full range via molecular docking. After the molecular models of PPD and phospholipid (Figure 7) were established, the 3D conformation of the complex was obtained from the optimization results. In view of the optimized drug loading dosage being 31.17% (m/m; the molar ratio of PPD/phospholipid was 1/1.34 in products), it could be considered that the molar ratio of PPD and phospholipid in the complex was approximately 1:1. Therefore, the interaction between one PPD molecule and one phospholipid molecule was studied in docking. When the relative position was determined, the inclusion relationship with the interpolated charge surface and the H-bond interactions could be presented. The results showed that the hydrophobic portion of PPD molecule was surrounded by two hydrophobic arms of the phospholipid molecule, and one of the hydrophilic –OH group formed a hydrogen-bond (H-bond) with the –P=O structure in the phospholipid (Figure 8). The docking free energy was −3.3 kcal·mol^−1^.

### 2.4. Equilibrium Solubility Studies

**Figure 9:**
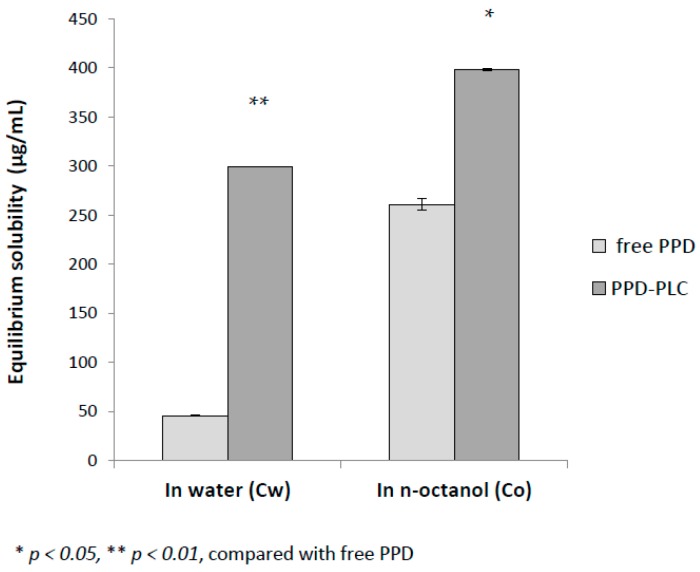
Equilibrium solubility of free PPD and PPD-PLC in water and in *n*-octanol at 25 °C (*n* = 3, mean ± SD).

Equilibrium solubility studies of free PPD and PPD-PLC were conducted in water (Cw) and in *n*-octanol (Co) (Figure 9). The average equilibrium solubility of PPD-PLC in water (299.40 ± 5.65 μg/mL) was 6.53-times that of free PPD (45.83 ± 0.57 μg/mL); whereas the solubility of PPD-PLC in *n*-octanol (398.45 ± 1.20 μg/mL) was 1.53-times that of free PPD (261.15 ± 0.35 μg/mL). This strongly suggested that the phospholipid complex could significantly promote the hydrophilicity (*p* < 0.01) and lipophilicity (*p* < 0.05) of the free drug.

### 2.5. In Vitro Dissolution Studies

**Figure 10:**
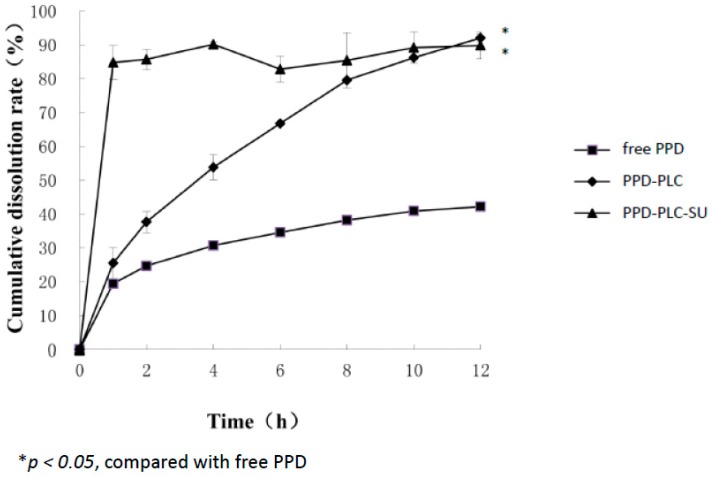
Comparative dissolution profiles of free PPD, PPD-PLC and PPD-PLC-suspension (SU) (*n* = 3, mean ± SD).

The dissolution curves of free PPD, PPD-PLC and PPD-PLC-SU are shown in Figure 10. Only 42.2% of the free PPD dissolved in 12 h could be distinguished, but nearly 80% of PPD-PLC had dissolved within the same time. The dissolution rate of PPD-PLC was obviously much higher than that of free PPD. However, approximately 85% of PPD-PLC-SU dissolved in the first hour, and the final dissolution extent remained approximately at 90%.

### 2.6. Stability Investigation

**Figure 11:**
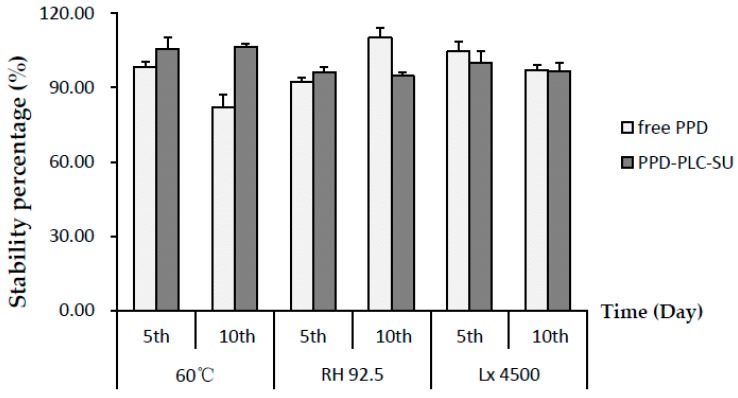
Stability investigation results of PPD and PPD-PLC-SU (*n* = 3, mean ± SD).

In our previous study [25], the stability of free PPD was revealed to be relatively sensitive to high temperature (≥40 °C). The results of the stability investigation on free PPD and its final formulation PPD-PLC-SU are shown in Figure 11. The DL percentage of PPD-PLC-SU (at the fifth and 10th day) were not influenced by high temperature (60 °C) and remained remarkably stable, compared with the percentage at zero days. Therefore, it indicated that the stability of PPD-PLC-SU was higher than free PPD under high temperatures. In high humidity, the stability of PPD-PLC-SU was shown to be a little lower than that of free PPD at the 10th day, which might be that the large amount of excipients in PPD-PLC-SU more easily become hygroscopic than free PPD when exposed to humid environments. It also showed that strong irritation had little effect on the stability of free PPD or PPD-PLC-SU at the fifth or 10th day. However, some results in the temperature-stability investigation slightly exceeded 100% (shown in Figure 11), which might be related to the moisture loss of the excipients in PPD-PLC-SU because of the drying process (60 °C) and measurement errors.

## 3. Discussion

During the property investigation of PPD in our previous research [25], the storage stability of PPD powder was investigated at different temperatures (40 and 60 °C), high humidity (RH 92.5% ± 5%), and strong illumination (4500 Lx ± 500 Lx). The free PPD powder degrades when stored or used in temperatures exceeding 40 °C, but remained unchanged under the other two conditions. This strongly suggested that PPD-PLC should be prepared at <40 °C to avoid content loss of the main drug during storage, which should be followed in the consequent forming process optimization. However, the stability investigation results indicated that phospholipid complex and dry suspension helped increase the stability of PPD at high temperature (60 °C), which indicated that the phospholipid complex might protect the complexed drug from being affected by high temperature, which might be related with the structure of PPD-PLC [52]. It also indicated that PPD-PLC could endure higher temperatures in the preparation or storage processes compared with free PPD. Additionally, the results detected in high humidity suggested that extra attention should be paid to the package condition in the production of PPD-PLC-SU, especially in moisture resistance.

A series of physicochemical methods were applied to characterize PLC formation. Similar to the observation of previous researchers in their cases of the PLC study [53], PPD and phospholipids had some interactions. The DSC results indicated that this interaction of the functional group (hydrophilic) of C–OH in PPD and phospholipids might contribute to form the complex with a hydrogen bond connection or van der Waals forces, which led to the hydrophilic effect enhancement and physiochemical stability of PLC. The PXRD diffraction pattern indicated that the crystallinity of PPD in PPD-PLC was reduced, which might be associated with the combination of PPD and phospholipids with a polar-end orientation, resulting in the change in its solid morphology. The shift from the crystalline state of free PPD to the amorphous in PPD-PLC might be related to the enhanced solubility of PPD in PPD-PLC, which was confirmed by the results of solubility determination. Additionally, the results of FTIR hinted that the nonpolar portions might combine with van der Waals forces. ^1^H-NMR spectroscopy highlighted that new compounds were not produced in the complex process. The results of molecular docking studies also indicated that the formation of the complex was an exothermic process, which was consistent with the DSC results. The two portions of hydrophobic and hydrophilic interactions matched the two new endothermal peaks, which appeared at 153.67 and 294.16 °C. Figure 8b shows that the H-bond between the –P=O of the phospholipid and –OH of PPD was formed with a length of 1.34 Å. This corresponded to the FTIR data in Figure 5, where the wide peak at the wavenumber of 3238.48 cm^−1^ was shifted to 3207.62 cm^−1^ with a weakened intensity. At the same time, the formation of intermolecular H-bond also affected the H-bond interaction between the two adjacent –OH groups in PPD molecule. This was also shown in the FTIR data at the wavenumbers of 3238.48 cm^−1^ and 1120.64 cm^−1^, where the peaks had disappeared.

Actually, a diffraction peak at about 20(2θ) could be observed in the PXRD pattern of the phospholipid (Figure 4B), although it was very tiny when compared with the distinguishable peaks of other PXRD patterns. In theory, the diffraction peaks of this kind are not expected to appear in PRXD detection, when phospholipids are of high purity (≥90%). However, the phosphatidylcholine content of the phospholipids used in this study (egg yolk lecithin, PL-100M, Kewpie Corporation, Tokyo, Japan) was about 80% (*w*/*w*), with a relatively low purity. As the analytical certificate reported, there was about another 17% “phosphatidylethanolamine” in this phospholipid. Therefore, in our opinion, this diffraction peak might be related with the existence of phosphatidylethanolamine. Some reports in recent years confirmed this inference, in which their results also showed that there were some tiny diffraction peaks in the XRD patterns of free phospholipids, when lecithin of relatively low content (<90%) of phosphatidylcholine was used. This phospholipid materials included Lipoid S75 [52] and Lipoid E80 [54]. Furthermore, this result in PXRD characterization also reminds us that phosphatidylcholine of high purity should be used in the study of the phospholipid complex, such as PC-98T (egg yolk lecithin, ≥98%, Kewpie Co., Tokyo, Japan) or Lipoid S100 [34,55] (soya phosphatidylcholine, ≥94%, Lipoid GmbH, Ludwigshafen, Germany).

It is generally known to us that three factors, including solubility, membrane permeability and first-pass effect, are the main factors that affect the oral absorption of drugs, in which solubility mainly affects the release rate of drugs; membrane permeability determines the transporting speed through the digestive tract epithelial cells and specific absorption site of drugs in the digestive tracts; and first-pass effects have a great impact on the absorption degree, including the gastrointestinal tracts and liver metabolism enzymes. For those poorly-soluble drugs, enhancement of their solubility might be one of the most effective ways to improve the oral bioavailability, which was also the focus in this paper.

Using PPD-PLC as the intermediate, PPD-PLC-SU was prepared with proper pharmaceutical formulation, and a dissolution study and storage stability investigations were then conducted. The results of the dissolution study revealed the significantly improved dissolution extent and rates (*p* < 0.05), which indicated that the amphiphilic phospholipid molecules might promote the dissolution of PPD. The hydrotropic effects were greatly magnified by the entrapment of PPD into PLC. Much of these amplification effects might be attributed to the superior dispersibility, amorphous form of the phospholipids complex and the combination of polar groups from PPD and excipients. We speculated that the hydrophobic functional group of PPD was close to the phospholipid molecules through quasi-stable bonding, and therefore, the nonpolar moiety of PPD was masked by the phospholipids, which was consistent with another study [56]. Chemically, phospholipid (also called lecithin) is an essential component of cell membranes with strong affinities. When the drug-PLC is formed, phospholipid, the drug carrier, promotes drug transfer from the hydrophilic environment to the lipophilic intestinal epithelial cell membrane into the cells and finally into the blood [57]. Moreover, phospholipid interacts with groups associated with certain adverse effects. Therefore, PLC technology is also expected to ultimately reduce the stimulation or side effects of these active substances [44]. In this study, we developed a type of PLC-incorporating PPD, an inferiorly-soluble, but biologically-active component extracted from herbal medicinal materials, which contained free PPD and phospholipids without any organic solvent. The phospholipids used in the study were of injection grade with an 80% (*w*/*w*) phosphatidylcholine content, which was demonstrated to be biologically safe, non-carcinogenic and non-mutagenic [58,59].

In the contemporary pharmaceutical community, PLC is often treated as an intermediate in the development of a final dosage form instead of a direct medical application because of its inferior stability. In this study, the dissolution curve of PPD-PLC-SU additionally disclosed the possibility of a quick-release formulation, which might be related to the excipients of the dry suspension. The results of the dissolution test showed that PPD-PLC-SU has rapid dissolution, compared with PPD-PLC. In our opinion, this is due to the advantage of the proposed SU formulation, which is composed with 79.17% Avicel CL611 (suspending agent) and 15.83% Poloxamer 188 (wetting agent). This result also confirmed the advantageous property of the dry suspensions formulation, as mentioned above. It can be expected that during the beginning of dissolution test, the PPD-PLC-SU powder was rapidly wetted because of the existence of wetting agent (Poloxamer 188), compared with the PPD-PLC powder. In addition, with the impeller agitation speed of 100 rpm, PPD-PLC-SU was well dispersed in the dissolution media and could be rapidly suspended with the assistance of the suspending agent (Avicel CL611). By contrast, although PPD-PLC also had improved dissolution, compared with free PPD powder, it did not have enough motive force to be moistened and suspended in the dissolution media within a short time. Therefore, PPD-PLC-SU presented more rapid dissolution, when compared with PPD-PLC. The patent on this formulation of the dry suspension was authorized by the Chinese State Intellectual Property Office in the year 2014 (ZL201210120958.5/CN102631322B). The combined application of two or more pharmaceutical technologies was observed to compound the effects. Other PLC-based dosage forms include nanoparticles [44,60], SMEDDS [60,61], solid dispersions [37,62], microspheres [37], micelles [57], pellets [63] and other forms [64,65]. The combination of PLC and nanoparticles was reported to considerably reduce the toxicity and enhance the pharmacological efficacy of the drug compound [44]. Some other nanoparticles were developed with PLC to successfully obtain sustained or controlled drug-delivery systems [64,66]. However, the majority of PLC applications involved the enhancement of water solubility or liposolubility, similar to the results of this study, and focused on improving the gastrointestinal absorption and oral bioavailability of drugs [67,68]. However, PLC technology cannot solve the oral absorption problems associated with all insoluble components, although drugs with a phenolic hydroxyl group reportedly have a relatively high EE [61].

## 4. Materials and Methods

### 4.1. Materials

PPD with a purity of ≥98% was purchased from Jingke Chemistry Technology Co., Ltd., Shanghai, China. Phospholipid (PL-100M) with a phosphatidylcholine content of 80% (*w*/*w*) was purchased from Shanghai Advanced Vehicle Technology Corporation, Shanghai, China. Avicel CL-611 was purchased from FMC Corporation, Philadelphia, PA, USA. Poloxamer 188 (Pluronic F68) was purchased from BASF Corporation, Ludwigshafen, Germany. All other chemical reagents were of analytical grade or higher.

### 4.2. PPD-PLC Preparation

**Figure 12:**
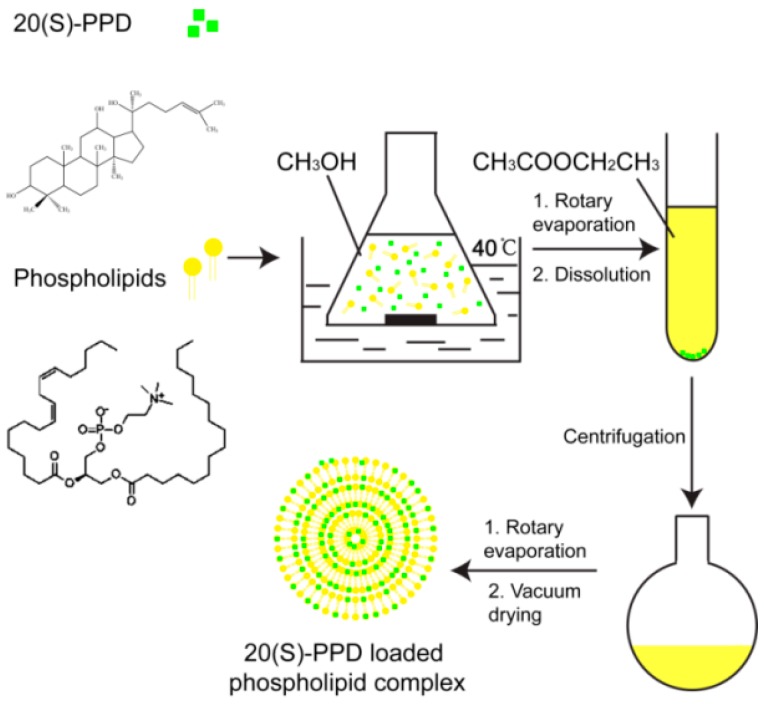
Scheme of the PPD-PLC preparation.

PPD-PLC was prepared using PPD and phospholipids through a slightly modified anti-solvent method [69]. The detailed scheme of the preparation is shown in Figure 12. Certain amounts of phospholipids and PPD were weighed (at weight ratios of 1:2, 5:6, 5:4, 5:3 and 2:1; g/g), placed in a flat-bottom triangle flask and dissolved in 20 mL of methanol, with PPD concentrations of 5.00, 8.17, 12.50, 16.83 and 20.00 mg/mL. The reactant mixtures were stirred using a magnetic stirrer (SHJ-4 thermostatic magnetic stirrer, Jintan Medical Instrument Co., Ltd., Jintan, China) at 40 °C and 1300 rpm for 1–5 h (1, 1.5, 3, 4.5 and 5 h). The resultant clear solution was subsequently dried, and another 10 mL of ethyl acetate were added to dissolve the residue, followed by sonication for 3 min. The resultant mixture was transferred to a 15-mL centrifuge tube and centrifuged (80-2 Centrifuge, Shanghai Medical Instruments Co., Ltd., Shanghai, China) for 15 min at 4000 r/m. The supernatant was dried under vacuum at 60 °C to remove the solvent residuals; following which, the resultant PPD complex products were collected and conserved in an amber-colored glass bottle at room temperature.

#### 4.2.1. Process Optimization of PPD-PLC

The process of PPD-PLC was optimized by using a CCD and RSA. According to the results of early preliminary experiments, the factors (independent variables) that had a relatively strong influence on the EE (dependent variables), phospholipid-to-drug ratio (*X*_1_, g/g), concentration of PPD in the reaction medium (*X*_2_, mg/mL) and reaction time (*X*_3_, h) were selected. An optimization procedure was performed by applying a CCD and RSA to systematically analyze the compound influence of these factors in a reduced number of trials. Briefly, in this design, three factors with five levels each were evaluated, and the trials were performed in all 20 possible combinations. EE was considered as the response variable. A statistical model that incorporated the linear effects (Equation (3)) and polynomial terms (Equation (4)) by using Design-Expert was used to evaluate the response. The models [70] are expressed as follows.

*Y* = *a*_0_ + *a*_1_*X*_1_ + *a*_2_*X*_2_ + *a*_3_*X*_3_ (3)

*Y* = *b*_0_ + *b*_1_*X*_1_ + *b*_2_*X*_2_ + *b*_3_*X*_3_ + *b*_11_*X*_1_^2^ + *b*_22_*X*_2_^2^ + *b*_33_*X*_3_^2^ + *b*_12_*X*_1_*X*_2_ + *b*_23_*X*_2_*X*_3_ + *b*_13_*X*_1_*X*_3_ (4)

where *Y* is the dependent variable (EE, %), *a*_0_ and *b*_0_ are the intercepts representing the arithmetic mean response of the 20 runs and *a_i_*, *b_i_* are the estimated coefficients for the factor *X_i_*. The main effects (*X*_1_, *X*_2_ and *X*_3_) represent the average results of changing one factor at a time from its minimum to maximum value. The interaction terms (*X*_1_*X*_2_, *X*_2_*X*_3_ and *X*_1_*X*_3_) revealed the response changes when two factors are changed simultaneously. The polynomial terms (*X*_1_^2^, *X*_2_^2^ and *X*_3_^2^) were included to investigate nonlinearity. The levels of the three independent variables and the composition of the CCD batches are presented in Table 3.

#### 4.2.2. Validation of Model Optimization

After the RSD on the results of CCD experiments, the optimal process parameters were obtained. Three batches of PPD-PLC were prepared under the optimized conditions to evaluate the optimization capability and validate the optimized model and then compared with the predicted valued calculated by the Design-Expert software. The bias was calculated using Equation (5).

Bias (%) = (predicted value − observed value)/predicted value × 100(5)

#### 4.2.3. Determination of Entrapment Efficiency

When a certain amount of prepared PPD-PLC was dispersed in 5 mL of ethyl acetate, both PPD-PLC and phospholipids were easily soluble in ethyl acetate; however, the free PPD materials remained essentially insoluble in ethyl acetate. Because of this solubility difference, methanol (a good solvent of PPD) was added to the precipitate (the unentrapped PPD), and the sample was centrifuged. The amount of free PPD was obtained by detecting the content of PPD in the resultant supernatant. Each experiment was performed in triplicate. The EE of PPD-PLC was determined using Equation (6).

(6) $$\mathrm{EE}(\%)=\frac{\text{Amount of total PPD added}-\text{Amount of unentrappe d PPD}}{\text{Amount of total PPD added}}\times 100$$

#### 4.2.4. Determination of DL

Approximately 50 mg of PPD-PLC powder were dissolved in 50 mL of methanol. After appropriate dilution, a 20-μL aliquot of the sample solution was injected into a high performance liquid chromatography (HPLC) system after filtration through a 0.45-μm membrane to determine the concentration of PPD in PPD-PLC. Subsequently, the amount of PPD in the samples was calculated. Each experiment was performed in triplicate. The DL of PPD-PLC was determined using Equation (7).

(7) $$\mathrm{DL}(\%)=\frac{\text{Amount of PPD in PPD}-\mathrm{PLC}}{\text{Amount of PPD}-\mathrm{PLC}}\times 100$$

#### 4.2.5. Method Validation of Content Determination

The content of PPD in the phospholipid complex was determined with HPLC [25]. Approximately 50 mg of the complex powder was dissolved in 50 mL methanol. The sample solution was prepared by properly diluting the sample with methanol. Next, a 20-μL aliquot of the sample solution was injected into an HPLC system (Agilent 1100, Agilent, Santa Clara, CA, USA) coupled with an Agilent Eclipse XDB-C18 column (4.6 mm × 150 mm, 5 μm). The mobile phase consisted of acetonitrile and water at a ratio of 88:12. The elution was performed at a flow rate of 1.0 mL/min and at 25 °C, and a detection wavelength of 203 nm was used.

Method validation included linearity, sensitivity, accuracy and precision. The linearity of the calibration curve was established by plotting the detected response area ratio versus the concentration of the standard solutions of different levels. It was established based on the injections of the standard solutions at concentrations within the range of 15.2–1015.6 μg/mL (15.2, 51.6, 162.5, 516.4 and 1015.6 μg/mL). Each of the solutions was injected in triplicate. A calibration curve equation was calculated for quantification of PPD using the peak area (*y*) and concentration (*x*). The determination coefficient (*r*) and linear dynamic ranges were determined. The LOD and LOQ were determined for sensitivity using the signal-to-noise method and expressed by the determinations at certain signal-to-noise ratios (*S*/*N* = 3 and 10), respectively. The accuracy of the method was evaluated though recovery experiments by spiking standard PPD into PPD-PLC samples at three levels (120%, 100% and 80%), with three replicates at each level (*n* = 3). The mixture samples were then treated with the aforementioned procedure and injected to an HPLC analyzer. The recovery was calculated as the ratio (%) of the determined spiked amount to the actual addition amount of PPD. In addition, the intra-day (*n* = 6) and inter-day (*n* = 5) precision studies for three standards (15.2, 162.5 and 1015.6 μg/mL) were investigated, respectively, where the results were expressed by the relative standard deviation (RSD).

### 4.3. Physiochemical Characterization of PPD-PLC

#### 4.3.1. DSC

The samples were loaded into aluminum pans separately with a 10 °C/min heating rate under a nitrogen atmosphere (50.0 mL/min) from 25–400 °C. The peak transition onset temperatures of the samples (free PPD, phospholipids, physical mixture and PPD-PLC) were determined using a differential scanning calorimeter (DSC Q2000, TA, New Castle, DE, USA). The free PPD, phospholipids and their physical mixture were used as controls.

#### 4.3.2. PXRD

The PXRD pattern of samples (free PPD, phospholipids, physical mixture and PPD-PLC) were acquired by increasing the diffraction angle (2θ) from 4°–40° at a scan rate of 0.04° (2θ/s) on an X-ray diffractogram (Bruker D8 Advance, Bruker, Karlsruhe, Germany) to investigate the physical state of the drug in PPD-PLC. The free PPD, phospholipids and their physical mixture were used as controls.

#### 4.3.3. FTIR

The samples were taken in KBr pellets using a Fourier-transformed infrared spectrophotometer (FTIR Presitage 21, Shimadzu, Kyoto, Japan). The spectra of the samples (PPD, phospholipids, physical mixture and PPD-PLC) were recorded in the 4000 cm^−1^–400 cm^−1^ region. The free PPD, phospholipids and their physical mixture were used as controls.

#### 4.3.4. ^1^H-NMR

The samples (free PPD, phospholipids, physical mixture and PPD-PLC) were dissolved in deuterium-substituted solvent and analyzed using a ^1^H-NMR spectrometer (AVANCE III UltraShield-Plus NMR Spectrometer, Bruker, Switzerland) at 400 MHz. The free PPD, phospholipids and their physical mixture were used as controls.

### 4.4. Molecular Docking

The molecular structure of PPD was extracted from the single crystal structure of 20-*O*-β-glucopyranosyl-20(*S*)-protopanaxadiol dehydrate obtained from the Cambridge Structural Database (Refcode: JEPJEU) and reconstructed. The molecular structure of the phospholipid was extracted from the crystal structure (PDB ID: 3B7Q) of yeast sec14 homolog sfh1 in complex with phosphatidylcholine of the RCSB PDB (Research Collaboratory for Structural Bioimformatics, Protein Data Bank) [71]. The molecular dynamics program GROMACS 4.0.5 was employed for performing energy minimization calculation. The docking program AutoDock Vina 1.1.2 [72] was used to perform the automated molecular docking calculation.

Before docking, the energy minimization protocol was executed to establish PPD and phospholipid models. The heavy atom of PPD with a highly rigid cyclic-structure was fixed. A flexible phospholipid molecule was then docked to it. This initial docking result with relatively low energy was used for the subsequent structure optimization.

In the minimization protocol, all of the models were put in the vacuum. The non-bond cutoff distance of 18.5 Å, spline width of 1.0 Å and buffer width of 0.5 Å were set. In the docking protocol, a Lamarckian genetic algorithm (LGA) in combination with a grid-based energy evaluation method was used for pre-calculating grid maps, according to the interatomic potentials of all atom types presented in the host and guest molecules, including the Lennard–Jones potentials for van der Waals interactions and Coulomb potentials for electrostatic interactions. A grid map of dimensions (50 Å × 50 Å × 50 Å), with a grid spacing of 0.375 Å, was placed to cover the PPD structure. With the help of AutoDockTools (Version 1.4.5, The Scripps Research Institute, La Jolla, CA, USA), the atomic partial charges were calculated by the Gasteiger–Marsili method [73]. The parameters used for the global search were an initial population of 50 individuals, with a maximal number of energy evaluations of 1,500,000 and a maximal number of generations of 50,000 as an end criterion. Other docking parameters were set at default values.

### 4.5. Equilibrium Solubility Studies

Equilibrium solubility was determined by using the supersaturated solution of free PPD and PPD-PLC in 5 mL of water or *n*-octanol in sealed glass containers at 25 °C. Each experiment was performed in triplicate. The liquid samples were agitated for 24 h and then centrifuged to remove excess PPD at 4000 r/m for 10 min. The supernatant was filtrated through a 0.45-μm membrane, and a 20-μL aliquot of the filtrate was injected into an HPLC system to be detected at 203 nm to determine the concentration of PPD.

### 4.6. PPD-PLC-SU Preparation

The produced PPD-PLC and Poloxamer 188 were ground over a 100-mesh sieve. According to our previous study [49], the produced PPD-PLC containing approximately 0.7 g of PPD, 30 g of Avicel CL611 and 6 g of Poloxamer were mixed in a three-dimensional mixer machine (T2F Turbular, Wabmachinery Corporation, Shenzhen, China). The resultant mixture was PPD-PLC-SU.

### 4.7. In Vitro Dissolution Studies

Dissolution studies were performed according to the specifications of the dissolution test apparatus reported [74]. The dissolution flasks were immersed in a water bath at 37 °C with 900 mL of dissolution medium (distilled water containing 0.25% Tween-80) and continuously stirred at 100 r/m. The free PPD, PPD-PLC and PPD-PLC-SU, each with an equivalent PPD amount of approximately 27 mg, were subsequently added into the stirred dissolution media. Afterwards, 5 mL of the sample solution in each cup were withdrawn and passed over 0.45-μm membranes at different time intervals (1, 2, 4, 6, 8, 10 and 12 h). After sampling, another 5 mL of fresh dissolution media were supplemented to each corresponding flask. Each experiment was performed in triplicate.

### 4.8. Stability Investigations

The stabilities of free PPD and PPD-PLC-SU were evaluated by calculating DL upon storage at a high temperature of 60 °C, high humidity (RH 92.5% ± 5%) at 25 °C and 4500 Lx ± 500 Lx illumination conditions. The samples were determined at the 5th and 10th day.

### 4.9. Statistical Analysis

All of the data are presented as the mean ± SD or mean. Multiple comparisons of the means (ANOVA) were used to substantiate statistical differences between groups, and Student’s *t*-test was used to compare two samples. Significance was tested at the 0.05 level of probability (*p*). Data analysis was performed with the SPSS software package (Version 19.0, IBM, Armonk, NY, USA) or Design-Expert (Version 8.0.6, Stat-Ease Inc., Minneapolis, MN, USA).

## 5. Conclusions

In this study, PPD-PLC was successfully designed and prepared with optimal process parameters, which were developed by applying CCD and RSD. The resultant products were characterized and evaluated to confirm the achievement of favorable physiochemical properties. The results of equilibrium solubility investigations strongly suggested that the phospholipid complex could significantly promote the hydrophilicity and lipophilicity of PPD. The PPD-PLC-SU was prepared with PPD-PLC as an intermediate. In addition, in vitro dissolution behaviors of PPD-PLC and PPD-PLC-SU were also investigated and the results revealed significantly improved dissolution extent and rates (*p* < 0.05). Molecular docking calculation combined with FTIR and DSC analyses revealed the possible interaction mechanism between PPD and phospholipids, and the mutual reflection of calculation and experiments strengthened the explanation of the mechanism. The results of stability investigations also showed that PPC-PLC possessed superior stability under high temperature, compared with free PPC. Therefore, the phospholipid complex, an efficient intermediate product in dry suspensions, makes PPD a possible candidate drug with great potential for clinical trials and application. Through enhancing the hydrophilicity and lipophilicity of free drugs, the absorption and bioavailability of PPD might be promoted effectively. However, the pharmacokinetic process and in vivo mechanism of PPD-PLC remain to be further investigated.

## Figures and Tables

**Table 1:** Results of central composite design batches (*n* = 3, mean ± SD).

No.	*X*_1_	*X*_2_	*X*_3_	*Y*/%
1	−1	−1	−1	53.77 ± 0.46
2	1	−1	−1	60.37 ± 5.00
3	−1	1	−1	59.87 ± 3.01
4	1	1	−1	65.73 ± 6.93
5	−1	−1	1	54.81 ± 2.36
6	1	−1	1	61.44 ± 6.13
7	−1	1	1	53.42 ± 4.62
8	1	1	1	65.21 ± 7.05
9	−1.732	0	0	45.33 ± 2.08
10	1.732	0	0	64.22 ± 2.98
11	0	−1.732	0	55.46 ± 0.77
12	0	1.732	0	57.54 ± 2.32
13	0	0	−1.732	58.24 ± 0.40
14	0	0	1.732	66.55 ± 4.58
15–20	0	0	0	54.90 ± 2.83

**Table 2:** ANOVA of response surface quadratic model.

Source	Sum of Squares	D*_f_*	Mean Square	*F*-Value	*p*-Value	Significance
Model	459.36	9	51.04	5.35	0.0075	significant
*X*_1_	288.91	1	288.91	30.26	0.0003	
*X*_2_	21.73	1	21.73	2.28	0.1623	
*X*_3_	6.49	1	6.49	0.68	0.4288	
*X*_1_ *X*_2_	2.44	1	2.44	0.26	0.6240	
*X*_1_ *X*_3_	4.44	1	4.44	0.47	0.5107	
*X*_2_ *X*_3_	10.31	1	10.31	1.08	0.3233	
*X*_1_^2^	0.86	1	0.86	0.090	0.7701	
*X*_2_^2^	10.30	1	10.30	1.08	0.3233	
*X*_3_^2^	121.18	1	121.18	12.69	0.0052	
Residual	95.47	10	9.55			
Lack of Fit	55.50	5	11.10	1.39	0.3638	not significant
Pure Error	39.97	5	7.99			
Corrected Total	554.83	19				

**Table 3:** Factor levels for the central composite design.

Factors	Level				
	−1.732	−1	0	1	1.732
*X*_1_ (g/g)	0.5	0.82	1.25	1.68	2
*X*_2_ (mg/mL)	5	8.17	12.5	16.83	20
*X*_3_ (h)	1	1.85	3	4.15	5
